# PULPe Best Paper Award 2025

**DOI:** 10.1177/17531934261468480

**Published:** 2026-08-09

**Authors:** Tobias Laurell, Anne Sophie Kruit, Mona I Winge

**Affiliations:** 1 Department of Clinical Science and Education, Södersjukhuset, Karolinska Institutet, Stockholm, Sweden; 2 Department of Hand Surgery, Södersjukhuset,Stockholm, Sweden; 3 Department of Plastic, Reconstructive and Hand Surgery, Radboud University Medical Center, Nijmegen, the Netherlands; 4 Hand and Microsurgical Unit, Division of Orthopaedic surgery, Oslo University Hospital, Oslo, Norway

Dear Editor,

The Paediatric Upper Limb Project Europe (PULPe) is a collaborative network dedicated to improving care for children with upper limb conditions. Given the rarity and heterogeneity of these conditions, high-quality research is both challenging and essential. The annual PULPe Best Paper Award aims to promote collaboration and recognize excellence in the field. For the 2025 awards, we reviewed articles published in leading journals including the *Journal of Hand Surgery (European)*, *Journal of Hand Surgery (American)*, *Journal of Children’s Orthopaedics*, *Journal of Bone and Joint Surgery*, *Hand Surgery and Rehabilitation* and the *Journal of Hand Therapy*. Two awards are granted – the best congenital upper limb paper and the best paediatric miscellaneous paper. Each paper was independently assessed based on novelty, methodological rigour and clinical relevance.We are pleased to announce the winners of the 2025 PULPe Best Paper Awards.

## Best congenital upper limb paper

Bohn DC, Price JN, Novotny SA, Sylvanus TS, Van Heest AE. Syndactyly release with skin graft or skin graft substitute: a within-subject controlled trial. J Hand Surg Eur. 2025, Vol. 50(10) 1339–1347.

This randomized, within-subject controlled trial assessed whether a skin graft substitute (hyaluronic acid matrix) can replace full-thickness skin grafts in syndactyly surgery. By using each patient as their own control, the authors minimized bias and interpatient variability. The comparison of results in 54 webs at 24 months showed no clinically relevant differences in scar quality between hyaluronic acid matrix and full-thickness skin grafting. The substitute reduced operative time and avoided donor-site morbidity, although it required prolonged wound care and was associated with more complications, such as matrix failure or infection. This methodologically strong study provides high-quality evidence to guide surgical decision-making in congenital hand reconstruction.

## Best paediatric miscellaneous paper

Hendrych J, Havránek P, Bayer M, Bayer M, Cepelik, M, Pesl. The effect of vitamin D on the speed and quality of pediatric fracture healing. J Child Orthop. 2025, Vol. 19 (1) 29-47.

This prospective randomized blinded study evaluated the role of vitamin D in paediatric fracture healing. Sixty-three Caucasian children with forearm or femoral shaft fractures were randomly divided into two groups. One group received vitamin D supplementation, which resulted in significantly faster and higher quality radiographical fracture healing compared with the second non-supplemented group. The study combined serial biochemical and blinded radiographic evaluation, and identified a high prevalence of vitamin D insufficiency at the time of injury. These findings support a routine assessment and supplementation of vitamin D in children with fractures, and have important clinical implications for paediatric trauma care.

We commend the authors for their scientific rigour and clinical relevance. Their work exemplifies the PULPe aim of improving outcomes for children through collaborative research. We also thank the *Journal of Hand Surgery (European Volume)* for supporting this initiative.

On behalf of the PULPe jury

